# Analytical validation and chromosomal distribution of regions of homozygosity by oligonucleotide array comparative genomic hybridization from normal prenatal and postnatal case series

**DOI:** 10.1186/s13039-019-0424-6

**Published:** 2019-03-06

**Authors:** Jiadi Wen, Kathleen Comerford, Zhiyong Xu, Weiqing Wu, Katherine Amato, Brittany Grommisch, Autumn DiAdamo, Fang Xu, Hongyan Chai, Peining Li

**Affiliations:** 1 Department of Genetics, Yale University School of Medicine, New Haven, CT 06520 USA; 2 Diagnostic Genetics Program, University of Connecticut, Storrs, CT 06269 USA; 3 Shenzhen Maternity and Child Healthcare Hospital, Shenzhen, Guangdong China; 4 PreventionGenetics, Marshfield, WI 54449 USA

**Keywords:** Regions of homozygosity (ROH), Single nucleotide polymorphism (SNP) calling, Array comparative genomic hybridization (aCGH)

## Abstract

**Background:**

Regions of homozygosity (ROH) are continuous homozygous segments commonly seen in the human genome. The integration of single nucleotide polymorphism (SNP) probes into current array comparative genomic hybridization (aCGH) analysis has enabled the detection of the ROH. However, for detecting and reporting biologically relevant ROH in a clinical setting, it is necessary to assess the analytical validity of SNP calling and the chromosomal distribution of ROH in normal populations.

**Methods:**

The analytical validity was evaluated by correlating the consistency of SNP calling with the quality parameters of aCGH and by accessing the accuracy of SNP calling using PCR based restriction enzyme digestion and Sanger sequencing. The distribution of ROH was evaluated by the numbers, sizes, locations, and frequencies of ROH from the collection of data from parental, postnatal, and prenatal case series that had normal aCGH and chromosome results.

**Results:**

The SNP calling failure rate was 20–30% with a derivative Log2 ratio (DLR) below 0.2 and increased significantly to 30–40% with DLR of 0.2–0.4. The accuracy of SNP calling is 93%. Of the 958 cases tested, 34% had no ROH, 64% had one to four ROH, and less than 1% had more than five ROH. Of the 1196 ROH detected, 95% were less than 10 Mb. The distribution of numbers and sizes of ROH showed no differences among the parental, pediatric and prenatal case series and test tissues. The chromosomal distribution of ROH was non-random with ROH seen most frequently in chromosome 8, less frequently in chromosomes 2, 6, 10, 12, 11 and 18, and most rarely seen on chromosomes 15, 19, 21 and 22. Recurrent ROH occurring with a frequency greater than 1% were detected in 17 chromosomal loci which locates either in the pericentric or interstitial regions.

**Conclusion:**

With a quality control parameter of DLR set at below 0.2, the consistency of SNP calling would be 75%, the accuracy of SNP call could be 93%, and the observed chromosomal distribution of ROH could be used as a reference. This aCGH analysis could be a reliable screening tool to document biologically relevant ROH and recommend further molecular analysis.

**Electronic supplementary material:**

The online version of this article (10.1186/s13039-019-0424-6) contains supplementary material, which is available to authorized users.

## Background

Copy number neutral segments showing continuous homozygosity with no intervening heterozygosity, termed ‘regions of homozygosity’ (ROH) or sometimes ‘runs of homozygosity’, were first identified using the denser genome-wide microsatellite scans by Broman in 1999 [1]. Other terms used in the literature included ‘long contiguous stretches of homozygosity’ which is interchangeable to ROH and ‘loss of heterozygosity’ or ‘absence of heterozygosity’ which could be copy number neutral or a deletion occurring as a somatic event in cancer [2]. ROH arise when identical haplotypes are inherited from each parent and thus a long tract of genotypes is homozygous. These autozygous segments may represent uniparental disomy (UPD), ancestral homozygosity due to linkage disequilibrium, or regions inherited from a more recent common ancestor that are identical by descent (IBD). Cousin marriage or inbreeding gives rise to such autozygosity. The detection of large ROH, UPD, and consanguinity by the presence of multiple ROH could have diagnostic implications in clinical laboratories performing single nucleotide polymorphism (SNP) microarray analysis [3, 4]. Genome-wide data reveal that ROH are universally common in human genomes even among outbred individuals [1, 5]. The numbers and sizes of ROH are believed to be a reflection of recombination rates and individual demographic history, while the homozygosity burden can be used to investigate the genetic architecture of complex diseases [6, 7].

Over the past decade, the validation and utilization of genomic microarray platforms in clinical cytogenetics has significantly increased the analytical resolution and diagnostic yield in prenatal and postnatal genetic evaluations [8, 9]. Subsequently, microarray analysis has been recommended as the first-tier genetic test for patients with developmental disabilities or congenital anomalies [10]. Today, microarray analyses using either array comparative genomic hybridization (aCGH) with copy number and SNP oligonucleotide probes or SNP genotyping microarray provide a genome-wide analysis at a kilo-base level resolution for the detection of copy number variants (CNV) and ROH [4, 10–15]. To detect and report biologically relevant ROH in a clinical setting, it is necessary to validate the technical procedures and understand the distribution of ROH in the outbred population. Before the publication of the ACMG guideline [16], the reporting practices among laboratories were highly variable due to the differences among testing platforms, threshold parameters, and laboratory policies. Factors such as the probe density, the probe distribution across the genome, and the quality of the SNP calling (including error rates) influenced the quality of ROH detection. Each laboratory interpreted results of homozygosity with their own discretion per the conflicting data in the literature regarding what cutoff level should be used [6, 17]. In this study, we assessed the analytical validity of Agilent’s oligonucleotide aCGH on detecting ROH by evaluating the consistency and accuracy of SNP calling. We also analyzed the numbers, sizes, locations, and frequencies of ROH from prenatal and postnatal normal case series. These results provided an important reference in detecting and reporting ROH findings for a better diagnostic practice to the patients.

## Material and methods

### Patient population

The ROH data were collected from aCGH studies performed on prenatal and postnatal case series at the Yale Clinical Cytogenetics Laboratory from January 2014 to December 2017 [18]. All cases showed normal aCGH and chromosome results and were considered to be ‘cytogenomically normal’ individuals. Four case series were organized based on prenatal or postnatal clinical indications and tested sample types. The normal parental case series included 142 healthy adults who were tested in a follow up parental study to rule out carriers of a balanced chromosomal rearrangement and to verify familial origin of variants of uncertain significance detected in their children. The pediatric case series included 500 cases with clinical indications of developmental/intellectual delay, autism spectrum disorders, intrauterine growth retardation (IUGR), multiple congenital anomalies, hearing loss, seizures, and other constitutional conditions. The aCGH and chromosome analysis were performed on peripheral blood specimens for parental and pediatric case series. The prenatal case series included 195 cases performed on chorionic villi samples (CVS) and 121 cases on amniotic fluid (AF) samples. Clinical indications for prenatal studies included advanced maternal age (> 35 years), suspected fetal anomaly, previous abnormal pregnancies and a family history of a hereditary disease. The parental, pediatric, prenatal CVS, and prenatal AF case series represented a full spectrum of constitutional cytogenomic analysis in the current clinical settings.

Additional criteria for data selection included the Derivative Log2 Ratio (DLR) spread and ROH size limitations. ROH data was collected only from cases that had a DLR equal to or less than 0.2, except for cases used for the analysis of SNP calling failure rate, in which the cases with the different range of DLR (< 0.2, 0.2–0.3, > 0.3) were included. The average SNP probes per megabase (Mb) genome is 19 probes, the arbitrary size cutoff of ROH is 1 Mb. Therefore, any ROH less than 1 Mb was excluded. This study also excluded cases with multiple ROH with a sum size greater than 3% of the genome size that could indicate a family relationship.

### DNA extraction and aCGH analysis

DNA was extracted from peripheral blood specimens, CVS and cultured amniocytes using the Gentra Puregene Kit (Qiagen, Valencia, CA) following standardized procedures adopted from the manufacturer’s instructions. The oligonucleotide aCGH analyses using Agilent’s SurePrint G3 Human Genome CGH + SNP microarray 180 K kit or the 400 K kit (Agilent Technologies, Inc., Santa Clara, CA) were performed as previously described [8]. The Agilent 180 K kit contains 110,712 CGH probes and 59,647 SNP probes, and the 400 K kit contains 292,097 CGH probes and 119,091 SNP probes (2 probes/SNP). The spatial resolution is an average of approximately 19 SNP probes per Mb of genome. The peripheral blood samples from parental and pediatric cases were analyzed with either the 400 K or the 180 K kit, and all prenatal samples (CVS and AF) were analyzed using the 180 K kit. This aCGH procedure can achieve 99% sensitivity and 99% specificity using a sliding window of five to seven contiguous oligonucleotides, indicating an analytical resolution of 100–150 Kb for the 180 K kit and 40–50 Kb for the 400 K kit [8]. The aCGH data were processed through the Agilent CytoGenomics Software to generate a report of detected CNV and ROH with genomic and chromosomal visualization and genomic coordinates following the UCSC genome browser GRCH37/hg19 assembly (http://genome.ucsc.edu/).

### Assessing the consistency and accuracy of SNP calling by aCGH

The readout of SNP alleles by Agilent Cytogenomics Software includes successful calling of homozygous and heterozygous alleles and failure calling of ‘NN’. The SNP calling failure rate was calculated by the number of ‘NN’ alleles divided by the total number of SNPs. It is thought that the SNP calling can be affected by the quality of fluorescent labeling of test and control DNAs and co-hybridization them onto the microarray. DLR from the test and control hybridization signals has been used as an important quality control parameter for aCGH. To assess the consistency of SNP calling, 30 cases each analyzed by 180 K and 400 K with DLR ranging from 0.14 to 0.4 were collected to analyze the correlation between SNP calling failure rate and DLR.

The SNP alleles in the Agilent CGH + SNP arrays locate in the recognition site AGCT by restriction enzyme Alu I and GTAC by RsaI. Polymerase chain reaction (PCR)-based restriction enzyme digestion and Sanger sequencing were used to assess the accuracy of SNP calling by Agilent CGH + SNP arrays. Five SNPs (rs3844608, rs8060511, rs8062853, rs216166 and rs10871449) were chosen and PCR primer pairs for each SNP were designed using Primer Express 3.0 (Additional file 1: Table S1). SNP calling by aCGH from 25 cases with DLR < 2.0 were further verified by fragment pattern from the digestion of PCR products by AluI or RsaI. When the SNP calling and enzyme digestion pattern showed inconsistent results, the allelic patterns were further analyzed using Sanger sequencing of the PCR product. Routine PCR was performed according to a standardized protocol for native Taq DNA polymerase (Invitrogen). Fragment analysis was run on Agilent 2100 Bioanalyzer (Agilent Technologies, Inc., Santa Clara, CA). Sanger sequencing was performed by submitting purified PCR products to the Yale Center of Genomic Analysis.

### Assessing the distribution of ROH in case series

An Excel file was used to collect data from each case series. The input data for each case included an anonymous case number, gender, age, sample type, clinical indication, aCGH and chromosome results, microarray platform, DLR measurement, and ROH detected. The number of ROH, the number of SNP probes hybridized to each ROH, the ROH size defined by genomic coordinates, and the chromosomal G-band location of ROH for each case were recorded. The numbers and sizes of ROH were compared among the four case series. To assess the chromosomal distribution of ROHs, the observed numbers and sizes of ROH and adjusted numbers and sizes by the genomic portion of each chromosome were analyzed. Frequencies of recurrent ROH at specific chromosomal loci were calculated by the number of cases with the recurrent ROH divided by total number of cases. The database Progenetix was used for data conversion and analysis [19]. The chromosomal location and genomic coordinate of each ROH were entered into the database and converted into schematic views that allowed a visualization and comparison of ROH frequencies.

### Statistics

One-way ANOVA analysis was used to compare the difference of SNP calling failure rate among samples with different ranges of DLR (http://vassarstats.net/anova1u.html). Chi-square analysis was performed to compare the difference among the case series for the number and size distribution of ROH by using the webtool: Chi-square Test Calculator (http://www.socscistatistics.com/tests/chisquare2/Default2.aspx).

## Results

### Analytical validity of aCGH on detecting ROH

**Fig. 1:**
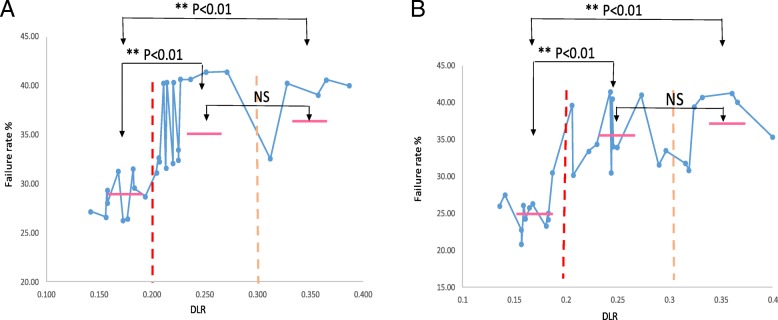
SNP calling failure rate in association with DLR. SNP calling data were extracted from **a**. Agilent 180 K aCGH/SNP array, and **b**. Agilent 400 K aCGH/SNP array. SNP calling failure rate increased significantly when DLR is greater than 0.2 (**, *p* < 0.01). No significant difference (NS) between samples of DLR 0.2–0.3 and DLR > 0.3

The density of SNP probes (defined by the number of probes per Mb) varied from 3 to 59 of detected ROH. The average number of SNP probes per Mb is 18 for both platforms which set analytical resolution arbitrarily as 1 Mb. The data of SNP calling on each SNP probe run by Agilent array 180 K and 400 K were exported from 30 cases with a range of DLR from 0.14 to 0.4. A total of 59,645 SNP probes were exported for each case run by the Agilent 180 K array, and 64,366 SNP probes for the 400 K array. The 30 cases were divided into three groups by DLR range of below 0.2, 0.2–0.3, and above 0.3. Of the 30 cases ran by 180 K, the mean SNP calling failure rate (NN calling rate) for 10 cases with DLR below 0.2 was 28.2%, for 15 cases with DLR range 0.2 to 0.3 was 36.5%, and for five cases with DLR above 0.3 was 38.5% (Fig. 1a). Of the 30 cases run by the 400 k array, the mean calling failure rate for 12 cases with DLR below 0.2 was 25.2%, for 11 cases with DLR range 0.2 to 0.3 was 35.3%, for seven cases with DLR above 0.3 was 37% (Fig. 1b). Statistical analysis showed significant difference between DLR < 0.2 and 0.2–0.3 or DLR > 0.3 (*p* < 0.01), but no difference between the DLR of 0.2–0.3 and DLR > 0.3 on both platforms. This result indicated that the DLR below 0.2 should be used as a quality control parameter to ensure consistency of SNP calling.

**Fig. 2:**
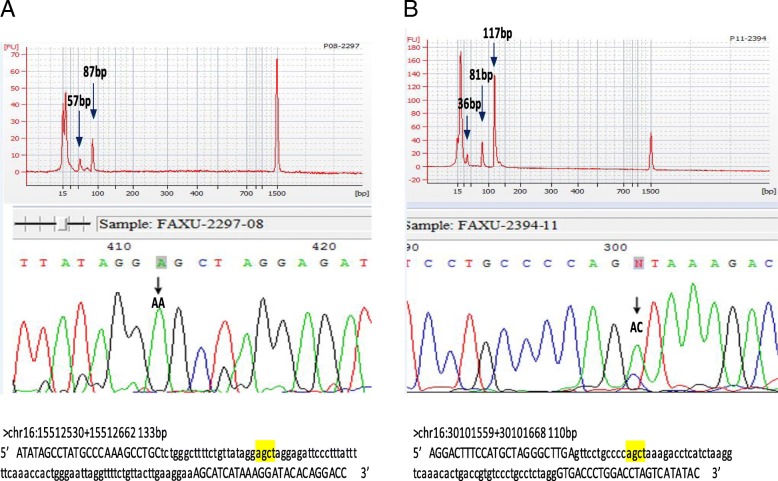
Confirmation of SNP calling by PCR-based restriction enzyme digestion and Sanger sequencing. **a**. Restriction enzyme digestion generated two PCR products of 57 bp and 87 bp, indicating an AA pattern which did not match the SNP call of GA. Sanger sequencing confirmed the PCR result of AA sequence (case number 16 in Additional file 1: Table S2); **b**. Restricted enzyme digestion generated three PCR products of 36 bp, 81 bp and 117 bp, indicating an AC pattern which did not match the SNP call of AA. Sanger sequencing confirmed the PCR result of AC sequence (case number 6 in Additional file 1: Table S2). The related primer sequence and enzyme cutting site are shown in the figure. Primer sequences are shown in capital letters

PCR was performed for the five selected SNP alleles on 25 cases with DLR below 0.2. Of these 125 SNPs tested, four SNP calling could not be confirmed by either PCR-restriction digestion or Sanger sequencing due to poor PCR amplification and were excluded. Of the remaining 121 SNPs, 112 SNPs showed consistent results from both aCGH SNP calling and direct PCR restriction enzyme digestion, but nine SNPs showed inconsistent results and were proved as false by Sanger sequencing on PCR products. The results of SNP callings from aCGH and patterns of PCR restriction enzyme digestion and Sanger sequencing are listed in Additional file 1: Table S2 and two verified false SNP callings by aCGH are shown in Fig. 2. Of the five SNPs tested, the accuracy of SNP calling by aCGH is 93% (112/121). It was noted that false callings occurred all in the two SNP alleles rs3844608 (four false in 25 callings) and rs8060511 (five false in 24 callings) but was not observed in the other three SNP alleles rs8062853, rs216166 and rs10871449. This observation suggested that some SNP loci may be prone to incomplete restriction enzyme digestion and induced false calling.

### Distribution and frequency of ROH

#### Numbers and sizes of ROH

**Fig. 3:**
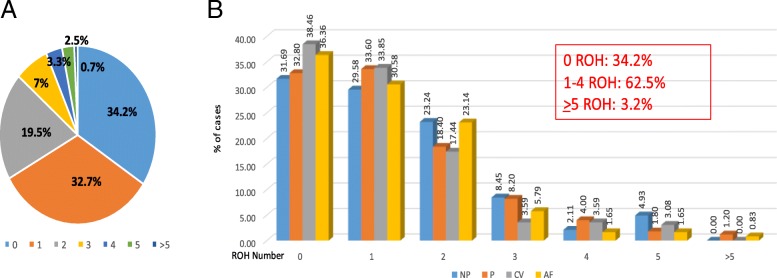
Distribution of ROH in the tested cases. **a**. A pie chart showing the percentage of different ROH counts (0, 1, 2, 3, 4, 5, and > 5) in the total of 958 tested subjects. **b**. A bar graph showing the comparison of ROH counts in different case series shown by different color (blue for NP, normal parents; orange for P, pediatric cases; grey for CV, prenatal CVS cases; and yellow for AF, prenatal AF cases)

A total of 958 cases were included in the study over the four years from 2014 to 2017. 142 were parental, 500 were postnatal/pediatric, 195 were prenatal CVS, and 121 were prenatal AF cases. All of them had normal karyotype and aCGH results. Cases with no ROH were detected in 31.7% of parental, 32.8% of pediatric, 38.5% of CVS, and 36.4% of AF cases. Cases with one ROH were detected in 29.6% of parental, 33.6% of pediatric, 33.9% of CVS, and 32.7% of AF cases. Cases with two ROH were detected 23.2% of parental, 18.4% of pediatric, 17.4% of CVS, and 23.1% of AF cases. Cases with three ROH were detected in 8.5% of parental, 8.2% of pediatric, 3.6% of CVS, and 5.8% of AF cases. Cases with four ROH were detected in 2.1% of parental, 4.0% of pediatric, 3.6% of CVS, and 1.7% of AF cases. Cases with five or more ROH were detected in 4.9% of parental, 3.0% of pediatric, 3.1% of CVS, and 2.5% of AF cases. There was no significant difference among the parental, pediatric, and prenatal case series, and no differences amongst the different tissue types (Additional file 1: Table S3, Fig. 3a). Therefore, of the 958 cases from the four-tested case series, the proportion of cases possessing no ROH, one to four ROH, and five or more ROH accounted for 34.2, 62.5, and 3.2% of cases, respectively (Fig. 3b).

**Fig. 4:**
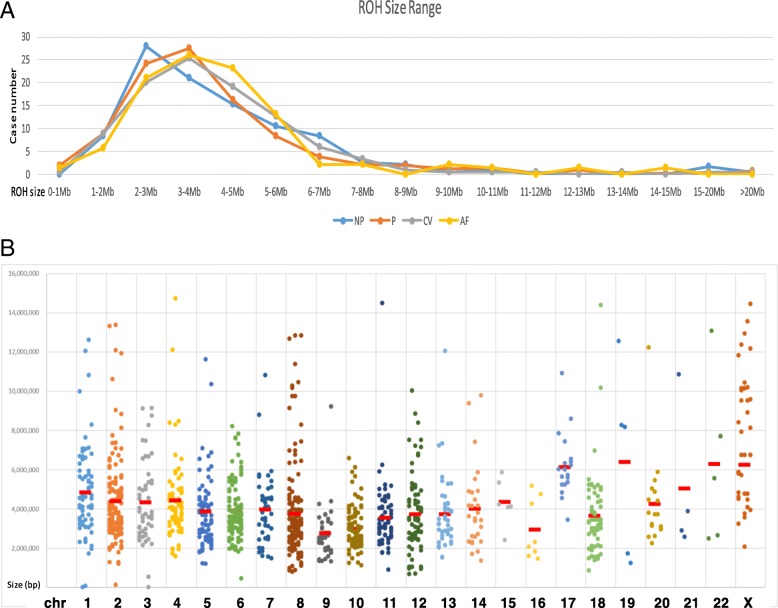
Size distribution of ROH in the tested cases. **a**. A linear graph showing the size distribution ranging by ROH size from 0 to > 20 Mb. Different sample groups were shown by different colors (blue/NP, parental cases; orange/P, pediatric cases; grey/CV, prenatal CVS; and yellow/AF, prenatal AF cases). No significant differences among groups (*p* > 0.05). **b**. Size distribution of ROH on each chromosome. Each dot represents one ROH. The red bars show the mean size of ROH on each chromosome

The sizes of detected ROH varied from 1 Mb to 20 Mb. ROH less than 5 Mb in size were noted in 73% of parental, 77% of pediatric, 74% of CVS, and 76% of AF case series, respectively. ROH less than 10 Mb in size were noted in 96% of parental, 94% of pediatric, 98% of CVS, and 97% of AF case series, respectively. The size distribution showed no significant differences among the four series (Fig. 4a). This result indicated that a cutoff value of 10 Mb can exclude more than 95% of ROH observed in normal cases.

#### Chromosomal location and recurrence of ROH in the genome

Of the 1196 ROH detected in the tested series, the sum of ROH sizes was 5163.3 Mb and the numbers and sizes of ROH distributed in each chromosome varied. The number of ROH observed ranged from 5 to 8 for chromosomes 15, 16, 19, 21 and 22, and from 18 to 150 for the other chromosomes. The average sizes of ROH ranged from < 3 Mb on chromosomes 9 and 16 to > 6 Mb on chromosomes 17, 19, 22 and X; the rest of the chromosomes had an average ROH size close to 4 Mb (Fig. 4b). Assuming a random distribution of ROH in each chromosome, the expected numbers and sizes of ROH on each chromosome were calculated per the actual portion of each chromosome in the whole genome times the total number or size of the detected ROH. Table 1 summarized the observed and expected numbers and sizes of ROH in each chromosome and showed a non-random distribution. Chromosomes 2, 6, 8, 10, 11 and 12 showed significant over-representation of both size and number of ROH, while chromosomes 3, 7, 14, 15, 16, 17, 19, 20, 21 and 22 showed under-representation. ROH was most prevalent in chromosome 8 (150/1196, 12.5%). The second most common location is in chromosome 2 (132/1196, 11.0%), followed by chromosomes 6 (98/1196, 8.2%), 10 (85/1196, 7.1%), 12 (76/1196, 6.4%), 4 (73/1196, 6.1%), 5 (73/1196, 6.1%), 11 (71/1196, 5.9%), 1 (68/1196, 5.7%), and 18 (59/1196, 4.9%). ROH was less likely to be seen in chromosome 16 with a frequency of 0.7% and was least likely to be observed in chromosomes 15, 19, 21, and 22 with a frequency of 0.4%.

**Table 1:** Chromosomal distribution of ROH

Chromosome	Chromosome size (bp)	% of genome	Observed ROH size (Mb)	Expected ROH size (Mb)	Observed ROH No.	Expected ROH No.
1	247,199,719	8	339.2	258.2	68	96
2	242,751,149	7.9	601.4	413.1	132	94
3	199,446,827	6.5	230.0	407.9	52	78
4	191,263,063	6.2	334.9	335.6	73	74
5	180,837,866	5.9	288.0	320.1	73	71
6	170,896,993	5.5	420.3	304.6	98	66
7	158,821,424	5.2	159.1	284.0	40	62
8	146,274,826	4.7	567.8	268.5	150	56
9	140,442,298	4.6	110.7	242.7	40	55
10	135,374,737	4.4	267.3	237.5	85	53
11	134,452,384	4.4	245.8	227.2	71	53
12	132,289,534	4.3	302.5	227.2	76	51
13	114,127,980	3.7	180.7	222.0	43	44
14	106,360,585	3.5	112.8	191.0	27	42
15	100,338,915	3.3	26.2	180.7	5	39
16	88,822,254	2.9	23.6	170.4	8	35
17	78,654,742	2.6	128.9	149.7	21	31
18	76,117,153	2.5	231.6	134.2	59	30
19	63,806,651	2.1	32.0	129.1	5	25
20	62,435,965	2	76.8	108.4	18	24
21	46,944,323	1.5	43.8	103.3	5	18
22	49,528,953	1.6	31.6	77.4	5	19
X	154,913,754	5	408.4	82.6	42	60
Total	3,079,843,747		5163.3		1196	

**Fig. 5:**
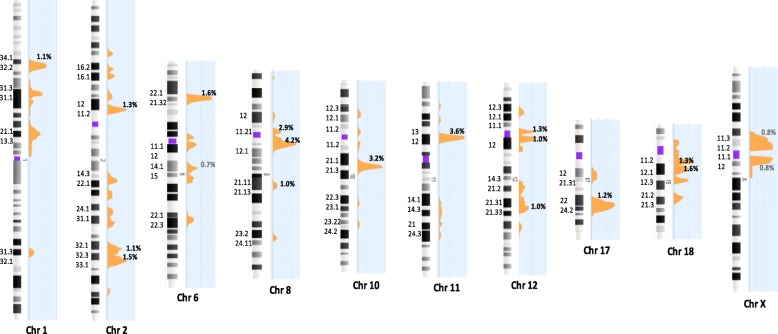
Schematic view of chromosomal regions and frequencies of the recurrent ROH. Recurrent ROH regions with a frequency greater than 1% are shown

Chromosomal regions harboring overlapping ROH in different cases were identified in the tested series. Those overlapping ROH with a frequency larger than 0.5% were considered as recurrent ROH in the genome. Of the total of 1196 ROHs, 47 chromosome regions of overlapping ROH were noted with a recurrent frequency of larger than 0.5% and 17 chromosomal regions of ROH were noted with a recurrent frequency larger than 1% (Additional file 1: Table S4). Of those 17 regions of recurrent ROH, 2p12p11.2, 8q11.1q12.1, 11p13p12, 12p11.1q12, and 18q11.2q12.1 are in the pericentric regions. Figure 5 showed the chromosomal regions with recurrent ROH. The most frequent regions harboring recurrent ROHs were 8q11.1q12.1 (chr8:46,940,022-58,936,071), 11p13p12 (chr11:35,538,690-41,944,671), and 10q21.1q21.2 (chr10:53,569,925-62,598,787) with a frequency of 4.2%, 3.6% and 3.2%, respectively. All recurrent ROH were observed in the pericentric regions or interstitial regions but not in the subtelomeric regions.

## Discussion

Implementation of CGH + SNP microarrays have enabled simultaneous interrogation of genomic DNA for CNV and copy neutral ROH. Multiple microarray platforms exist and vary in the placement and density of the SNP probes. Laboratories that perform this microarray analysis should establish quality control and quality assurance measures and reference values for each platform in the technical procedures and reporting practices. In this study, we proved that the current Agilent CGH + SNP 180 K and 400 K microarray platforms showed a consistent 73–75% SNP calling rate with DLR below 0.2, and the accuracy of SNP calling was approximately 93%. The SNP calling rate decreased significantly to 60–70% when the DLR ratio was greater than 0.2 and 0.3. According to the manufacturer’s quality control metrics parameters, DLR < 0.2 was considered as excellent, DLR in the range of 0.2–0.3 was considered as a “good run” while DLR greater than 0.3 was classified as “to be evaluated”. The SNP calling rate showed no difference from the DLR 0.2–0.3 and greater than 0.3 but a clear difference from DLR below 0.2. Therefore, to ensure consistency and accuracy in SNP calling, DLR below 0.2 should be used a cutoff value for reporting ROH. In practice, it is reasonable that DLR below 0.2 for current microarray platforms can be considered as a reliable tool for screening of ROH and reporting of CNV and ROH. When the DLR is 0.2~0.3, the SNP calling could be used to confirm CNV reading but only the CNV is to be reported, and when the DLR > 0.3, the case should be repeated.

It has been recognized that the detection of one or more ROH is not an abnormal finding in apparently normal individuals of an outbred population. ROH arise from the two brought-together ancestral haplotypes. Longer haplotypes are inherited from recent common ancestors whereas shorter haplotypes are inherited from distant ones. Different population histories give rise to divergent distributions of long and short ROH [6]. Although the numbers and sizes of ROH vary dramatically among published data, most of the studies showed that normal outbred populations rarely have ROH above 10 Mb and commonly have smaller ROH of less than 4~5 Mb [20, 21]. This occurs across all populations and is termed ancestral ROH. Our results from a total of 958 tested cases showed that 34% of cases had no ROH, 66% had at least one ROH, and 94% of this tested population had less than 3 ROH. Of the 1196 ROH detected, 70% of them had a size of less than 5 Mb and 95% of ROH were less than 10 Mb. These results were consistent with the published data.

ROH distribution over the chromosomes was found to be nonrandom in mammals, including humans [21–24]. ROH are more common in regions of high linkage disequilibrium (LD) and low recombination [5] as well as regions of low genetic diversity [21]. In our study, the most frequent region with ROH is the pericentromeric region of chromosome 8. Indeed, most of the regions that harbor a recurrent ROH are pericentromeric regions, which in theory should have a low recombination rate. Furthermore, the larger average size on the X chromosome also indicated low recombination frequency on the X chromosome through generations. Most chromosomes have a similar average ROH size of close to 4 Mb, so this may reflect the fact that there are no marked differences among chromosomes in the concentration of recombination hotspots [25]. Our data reported that, among the 1196 ROH greater than 1 Mb, seventeen overlapped regions were recurrently identified with a frequency of greater than 1%. The presence of recurrent ROH in the outbred population indicated that these ROH may represent an ancestral ROH with insignificant or limited clinical implications.

In our study, some chromosomes showed a very low frequency in presenting an ROH, such as chromosomes 15, 16, 19, 21 and 22. The underlying mechanism is unclear. Populations with both reduced effective population size in the past and recent inbreeding have the greatest burden of ROH; on the contrary, inbreeding depression could be a cause of eliminating the ROH in a certain chromosome. Studies showed that ROH are more enriched for homozygous deleterious variants than for non-deleterious variants [26], so that ROH are important reservoirs of homozygous deleterious variations [27]. In theory, inbreeding increases the chance of rare variants (including deleterious variants) to be in a homozygous state, and if the recessive variants are fatal, the inbreeding will in fact purge the recessive alleles from the population. Over time, this may result in fewer ROH in certain regions on certain chromosomes. We do not know if this could explain our findings, and it would be worthwhile to further investigate the rare, deleterious and fatal recessive variants on these chromosomes.

The cumulative extent of ROH in any given case is determined by multiple factors such as parental relatedness, ethnicity, recombination events, and chromosomal aberrations. The significance of such findings and deriving clues towards molecular testing and genotype–phenotype correlations necessitates critical evaluation of the microarray data in each individual case [28]. Wierenga et al. [29] reported variance in total ROH based on different size thresholds and illustrated the importance of threshold determination. The variation in cutoff values of ROH reported in the literature was reflected in the research laboratory’s reporting policy. In 2013, ACMG published a guideline regarding the documentation of suspected consanguinity by genomic testing [16]. From the size distribution detected by the current Agilent CGH + SNP array platforms, 10 Mb is a reasonable cutoff level to report the incidental finding of a single ROH. In addition, the recurrent ROH identified in our study indicated that these ROH may represent an ancestral ROH and should be considered as a reference set of “benign or likely benign polymorphic” ROH and should not be included in the reports. However, caution should be applied to the gene content especially disease-causing dominant or recessive gene mutations within these recurrent ROH. These cutoff settings should avoid missing important clinical information that may impart significant clinical consequences, but at the same time minimize the chance of overestimating it which may cause dramatic burden for the clinicians and genetic counselors, as well as to the patient’s family, both emotionally and financially.

## Conclusion

In the past decade, microarray testing for the assessment of genomic copy number imbalances in clinical laboratories has grown quickly. The detection of UPD, consanguinity, and isolated ROH has been an integral part of genomic analysis using many available platforms. The present study assessed the consistency and accuracy of SNP calling with the current Agilent CGH + SNP platforms and observed a nonrandom chromosomal distribution of ROH from parental, pediatric and prenatal case series. Based on the reference values of DLR below 0.2, 75% of SNP calling rate, and 93% accuracy of SNP calling, the CGH + SNP 180 K and 400 K platforms could be considered as a reliable method to screen for ROH larger than 10 Mb. If clinically indicated, correlating gene content of ROH with mutation testing results and patient phenotypes should be recommended.

## Additional file

Additional file 1: **Table S1.** PCR primer for selected SNPs. **Table S2.** Confirmation of SNP calling by PCR/Sequencing. **Table S3.** ROH distribution. **Table S4.** Frequencies of recurrent ROH. (DOCX 78 kb)

## Abbreviations

aCGH: Array comparative genomic hybridization
AF: Amniotic fluid
CNV: Copy number variants
CVS: Chorionic villi samples
DLR: Derivative Log2 Ratio
IBD: Identical by descent
LD: Linkage disequilibrium
ROH: Regions of homozygosity
SNP: Single nucleotide polymorphism
UPD: Uniparental disomy
