# Supplementary material for: Analytical validation and chromosomal distribution of regions of homozygosity by oligonucleotide array comparative genomic hybridization from normal prenatal and postnatal case series
Source: Mol Cytogenet. 2019 Mar 6;12:12. doi: 10.1186/s13039-019-0424-6 (PMC6404290; doi:10.1186/s13039-019-0424-6)
Supplement: Supplementary file 1 — Table S1. PCR primer for selected SNPs. Table S2. Confirmation of SNP calling by PCR/Sequencing. Table S3. ROH distribution. Table S4. Frequencies of recurrent ROH. (DOCX 78 kb) [file 13039_2019_424_MOESM1_ESM.docx]

Supplemental Table 1: PCR primer for selected SNPs

Supplemental Table2. Confirmation of SNP calling by PCR/Sequencing

Supplemental Table 3: ROH distribution

Supplemental Table 4: Frequencies of recurrent ROH
